# Safety and Pharmacokinetics of CXCR4 Peptide Antagonist, LY2510924, in Combination with Durvalumab in Advanced Refractory Solid Tumors

**DOI:** 10.1089/pancan.2019.0018

**Published:** 2020-03-12

**Authors:** Mark H. O'Hara, Wells Messersmith, Hedy Kindler, Wei Zhang, Celine Pitou, Anna M. Szpurka, Dan Wang, Sheng-Bin Peng, Burkhard Vangerow, Anis A. Khan, Mythili Koneru, Andrea Wang-Gillam

**Affiliations:** ^1^ Division of Hematology/Oncology, Abramson Cancer Center, University of Pennsylvania, Philadelphia, Pennsylvania.; ^2^ Medicine-Medical Oncology, School of Medicine, University of Colorado, Denver, Colorado.; ^3^ Gastrointestinal Oncology, The University of Chicago Medicine, Chicago, Illinois.; ^4^ Eli Lilly and Company, Indianapolis, Indiana.; ^5^ Eli Lilly and Company, Windlesham, United Kingdom.; ^6^ Formerly with Eli Lilly and Company, Indianapolis, Indiana.; ^7^ AstraZeneca, Gaithersburg, Maryland.; ^8^ Department of Medicine, Molecular Oncology, Washington University School of Medicine, Washington University Medical School, St. Louis, Missouri.

**Keywords:** CXCR4, durvalumab peptide antagonist, LY2510924, refractory solid tumor

## Abstract

**Purpose:** This was an open-label phase 1a study assessing the maximum tolerated dose (MTD), safety, and tolerability of CXCR4 peptide antagonist, LY2510924, administered in combination with durvalumab in patients with advanced refractory solid tumors.

**Methods:** Patients received LY2510924 at 20, 30, or 40 mg subcutaneous (SC) once daily in combination with durvalumab at 1500 mg intravenously (IV) on day 1 of each 28-day cycle. The primary objective was to assess the MTD and safety of LY2510924 SC daily in combination with durvalumab in patients with advanced (metastatic and/or unresectable) solid tumors. Secondary objectives included pharmacokinetics (PK) and the antitumor activity of LY2510924 in combination with durvalumab. Exploratory objectives were biomarker analysis, including pharmacodynamic markers, relevant to LY2510924 and durvalumab, including immune functioning, drug targets, cancer-related pathways, and the disease state.

**Results:** Nine patients (three each at 20, 30, and 40 mg) were enrolled in the study (eight patients with pancreatic cancer and one patient with rectal cancer). The majority of patients completed one or two cycles (100.0% ≥ 1 cycle; 88.9% ≥ 2 cycles) of LY2510924 and durvalumab. No dose limiting toxicities were reported. Most common (>10%) treatment-emergent adverse events were injection-site reaction (44.4%), fatigue (33.3%), and increased white blood cell count (33.3%). PK parameters for combination were similar to those reported in previous studies when given as monotherapy. Best overall response of stable disease was observed in four (44.4%) patients and one patient had unconfirmed partial response.

**Conclusion:** The recommended phase 2 dose is 40 mg SC once-daily LY2510924 in combination with durvalumab 1500 mg IV and showed acceptable safety and tolerability in patients with advanced refractory tumors.

## Introduction

Cancer metastasis is the major cause of cancer morbidity and mortality, and accounts for ∼90% of cancer deaths. However, effective treatment options for advanced cancer are limited.^1^ The chemokine (C-X-C motif) receptor 4 (CXCR4) and its only known ligand, α-chemokine stromal cell-derived factor-1 (SDF-1, also known as CXCL12), play an important role in the regulation of organ-specific metastasis, as well as in tumor growth, invasion, survival, and angiogenesis.^2^ CXCR4 is functionally expressed or overexpressed in a variety of solid tumors, lymphomas, and chronic lymphocytic leukemias.^3,4^ In addition, CXCR4 and the SFD-1 axis also play a critical role in leukocyte trafficking, T cell infiltration, and immune response.^5^

Pre-clinical and clinical studies of agents that target CXCR4 have demonstrated mobilization of immune cells and hematopoietic stem cells (HSCs) into peripheral blood by altering cell homing, retention, and release from the bone marrow compartment. The existing pre-clinical and emerging clinical data indicate CXCR4 to be an attractive target for antitumor drug development.^5,6^

LY2510924 is a potent, selective peptide antagonist of CXCR4 both *in vitro* and *in vivo*. In several cancer xenograft models utilizing cell lines that express high levels of CXCR4, LY2510924 has demonstrated dose-dependent inhibition of tumor growth and metastasis.^7^ Previously, in a phase 1 trial, LY2510924 was administered at doses ranging from 1 to 30 mg as a daily subcutaneous (SC) injection.^8^ At a dose of 30 mg/day, two patients experienced dose-limiting toxicity (DLT) defined as grade 3 increase in absolute neutrophil count (ANC) >25,000 cells/μL for >5 days.^8^ In another randomized phase 2 trial, the addition of LY2510924 20 mg SC to sunitinib in the first-line treatment of metastatic renal cell carcinoma (RCC) was well tolerated.^9^ In all these clinical studies, administration of LY2510924 was associated with a significant increase of cluster of differentiation (CD34+) HSCs and leukocytes in circulation.^8,9^

Durvalumab is a selective, high-affinity, human immunoglobulin G1 monoclonal antibody that blocks programmed death ligand 1 (PD-L1) binding to programmed death 1 (PD-1) and CD80, allowing T cells to recognize and kill tumor cells.^10–12^ While these inhibitors have effects in various solid tumors,^13,14^ PD-1 or PD-L1 inhibition has limited or no efficacy as a single agent in pancreatic cancer,^15,16^ possibly, in part, due to lack of immune cell infiltration in the tumor microenvironment.^17^ Due to the critical role of CXCR4 and SDF-1 in leukocyte trafficking and T cell infiltration, we hypothesize that a CXCR4 inhibitor, when combined with anti PD-L1 or anti PD-1 therapy, may have the potential to enhance effects of PD-1/PD-L1 inhibitors. Indeed, CXCR4 inhibition yields increased infiltration of T cells into tumors and tumor regression in an *in vivo* pancreatic cancer mouse model.^18^

Here, we report the data from an open-label phase 1a study assessing the safety and tolerability of LY2510924 in combination with durvalumab.

## Methods

### Study design

This study was an open-label, phase 1a, dose-escalation trial evaluating the safety and tolerability of LY2510924 administered in combination with durvalumab in patients with advanced refractory solid tumors (ClinicalTrials.gov Identifier: NCT02737072). The study protocol was approved by institutional review boards/ethics committees before initiation, and conducted in accordance with the Declaration of Helsinki; patients provided written informed consent before entering the study. The primary objective was to assess the maximum-tolerated dose and safety of LY2510924 in combination with durvalumab in patients with advanced solid tumors. Secondary objectives included pharmacokinetics (PK) and the antitumor activity. Exploratory objectives included pharmacodynamic (PD) assessments of mobilization of CD34+ cells, immune cell subtyping in blood, and PD-L1 expression in tumor tissue.

### Patients

Patients aged 18 years or older with a confirmed diagnosis of advanced solid tumor after failure of standard-of-care therapy(s) were included in the trial. Patients had at least one measurable lesion assessable using standard techniques by Response Evaluation Criteria in Solid Tumors (RECIST) v 1.1. Additional eligibility criteria included the following: adequate organ function, an Eastern Cooperative Oncology Group (ECOG) performance status (PS) of 0 or 1, and an estimated life expectancy ≥12 weeks.

Patients were excluded from the study if they had active autoimmune disorders or prior severe autoimmune or inflammatory disorders requiring immunosuppressive treatment. Patients requiring escalating or chronic supraphysiologic doses of corticosteroids (>10 mg/day of prednisone or an equivalent corticosteroid) for control of their disease or immunosuppressive agents were also excluded; in addition, patients with prior therapy with an anti-PD-1, anti-PD-L1, anti-PD-L2, or anti-cytotoxic T lymphocyte-associated antigen-4 antibody or any other antibody or drug specifically targeting T cell costimulation or checkpoint pathways.

### Study dose and treatment

Patients received LY2510924 at 20, 30, or 40 mg SC once daily in combination with durvalumab at 1500 mg, administered intravenously (IV) on day 1 of each 28-day cycle. The dose range of LY2510924 was selected based on the overall clinical information from three prior completed studies CXAA, CXAB (RCC), and CXAC (small-cell lung cancer).^6^ Lilly proposed to increase the predefined ANC threshold criteria to 75,000 cells/mL which was a limiting factor in the first-in-human CXAA study.

Regarding the durvalumab dose justification, a fixed dose of 1500 mg every 4 weeks (Q4W) [equivalent to 20 mg/kg Q4W] instead of every 2 weeks (Q2W) dosing was used, given a similar area under curve (AUC), modest differences in median peak and trough levels at steady state, and ease of administration.

### Safety assessment

Safety was assessed by monitoring adverse events (AEs), including severity, seriousness, and the possible relation to study drug, dose adjustments, DLTs, clinical laboratory test results, vital signs, electrocardiogram readings, ophthalmological assessments, and dermatological evaluations. All AEs observed in the study were graded using the Common Terminology Criteria for Adverse Events (CTCAE) version 4.03.

### Efficacy assessment

Overall response rate, duration of response, and duration of stable disease were evaluated for every two cycles. Both tumor markers and evaluation of PS by the ECOG scale were also used as response assessment.

### Biomarkers/PD assessment

Blood collection and tumor biopsies were conducted to assess PD effect and biomarker levels whenever possible. The primary PD biomarker was to quantify LY2510924 stimulatory effects on the mobilization of CD34+ cells as an indirect reflection of CXCL12/CXCR4 axis inhibition. Immunophenotyping for CD34+ cells was performed using flow cytometry on samples obtained at baseline (within 7 days of day 1 in cycle 1) and at multiple time points while on study. In addition, the blood samples to identify and determine the percentages and absolute counts of T, B, and natural killer (NK−) cells as well as the CD4+ and CD8+ subpopulations of T cells expressing Ki67 and HLA-DR markers were obtained at baseline and while on study. In addition, ANC and absolute lymphocyte counts were obtained from a standard local blood test.

Tumor tissues at baseline (obtained from a newly obtained core or excisional biopsy of tumor lesion or archived biopsy) were tested for PD-L1 expression using the Ventana PD-L1 (SP263) immunohistochemistry (IHC) assay on an automated BenchMark ULTRA platform, using the anti-human PD-L1 rabbit monoclonal antibody. PD-L1 expression on tumor cells was calculated as the proportion of cells expressing PD-L1 at any intensity above background staining. The data analysis was descriptive.

### Pharmacokinetic assessments

Blood samples were drawn from all patients for the assessment of LY2510924 in plasma and durvalumab concentrations in serum at prespecified time points. The PK of LY2510924 was assessed through intensive sampling, and the PK parameter estimates were calculated by standard noncompartmental methods of analysis. PK data for durvalumab were summarized by sampling time points, and a formal noncompartmental analysis was not conducted. Samples were analyzed using a validated liquid chromatography with tandem mass spectrometry. Summary statistics was tabulated for the PK parameters of LY2510924 and durvalumab by dose and study day.

## Results

### Patients

A total of nine patients (three patients each in LY2510924 at 20, 30, and 40 mg) were enrolled in the study (eight patients with pancreatic cancer and one patient with rectal cancer). All the patients were white, 66.7% were male and 55.6% had an ECOG PS of 1 (Table 1). The median age of the patients was 53 years (range 33–77). The majority of the patients (*n* = 6; 66.7%) had received ≥3 prior regimens. All the patients discontinued study treatment due to progressive disease. The postdiscontinuation follow-up ended as a result of patient death due to PD (*n* = 6, 66.7%) and lost to follow-up (*n* = 3, 33.3%).

**Table 1.** Patient Baseline Characteristics

Characteristics	N (%)
Age, years	
Median (range)	53 (33–77)
<65 Years	7 (77.8)
≥65 Years	2 (22.2)
Sex	
Male	6 (66.7)
Female	3 (33.3)
Race	
White	9 (100)
ECOG PS	
0	4 (44.4)
1	5 (55.6)
Tumor site	
Pancreatic	8 (88.8)
Rectal	1 (11.1)
No. of prior therapies	
1	0 (0)
2	3 (33.3)
≥3	6 (66.7)
≥1 Prior systemic therapy	
Adjuvant	4 (44.4)
Neoadjuvant	5 (55.5)
Locally advanced	1 (11.1)
Metastatic	7 (77.8)

ECOG, Eastern Cooperative Oncology Group; PS, performance status.

### Extent of exposure

The majority of patients completed one or two cycles (100.0% ≥ 1 cycle; 88.9% ≥ 2 cycles) of LY2510924 and durvalumab, with a median duration of treatment of 102 days for LY2510924 and 110 days for durvalumab.

### Safety

No DLTs were reported in this study at any of the dose levels, and there were no AEs resulting in death or discontinuation from the study treatment. All patients (*n* = 9) experienced ≥1 treatment-emergent adverse event (TEAE) possibly related to the study treatment. The most common (>10%) TEAEs related to study treatment were injection-site reaction (*n* = 4; 44.4%), fatigue (*n* = 3; 33.3%), and increased white blood cell count (*n* = 3; 33.3%) (Table 2).

**Table 2.** Most Common (≥10%) Treatment-Emergent Adverse Events Related to Study Treatment

	DURV1500-LY 20 mg/day (N = 3)^a^			DURV1500-LY 30 mg/day (N = 3)^a^			DURV1500-LY 40 mg/day (N = 3)^a^			Total (N = 9)		
Event (n, %)	Grade 1–2	Grade ≥3	Any grade	Grade 1–2	Grade ≥3	Any grade	Grade 1–2	Grade ≥3	Any grade	Grade 1–2	Grade ≥3	Any grade
Injection-site reaction	1 (33.3)	0 (0)	1 (33.3)	1 (33.3)	0 (0)	1 (33.3)	2 (66.7)	0 (0)	2 (66.7)	4 (44.4)	0 (0)	4 (44.4)
Fatigue	0 (0)	0 (0)	0 (0)	2 (66.7)	0 (0)	2 (66.7)	1 (33.3)	0 (0)	1 (33.3)	3 (33.3)	0 (0)	3 (33.3)
White blood cell count increased	2 (66.7)	0 (0)	2 (66.7)	1 (33.3)	0 (0)	1 (33.3)	0 (0)	0 (0)	0 (0)	3 (33.3)	0 (0)	3 (33.3)
Decreased appetite	0 (0)	0 (0)	0 (0)	2 (66.7)	0 (0)	2 (66.7)	0 (0)	0 (0)	0 (0)	2 (22.2)	0 (0)	2 (22.2)
Back pain	0 (0)	0 (0)	0 (0)	1 (33.3)	0 (0)	1 (33.3)	1 (33.3)	0 (0)	1 (33.3)	2 (22.2)	0 (0)	2 (22.2)
Hypothyroidism	0 (0)	0 (0)	0 (0)	1 (33.3)	0 (0)	1 (33.3)	1 (33.3)	0 (0)	1 (33.3)	2 (22.2)	0 (0)	2 (22.2)
Constipation	0 (0)	0 (0)	0 (0)	2 (66.7)	0 (0)	2 (66.7)	0 (0)	0 (0)	0 (0)	2 (22.2)	0 (0)	2 (22.2)
Nausea	0 (0)	0 (0)	0 (0)	2 (66.7)	0 (0)	2 (66.7)	0 (0)	0 (0)	0 (0)	2 (22.2)	0 (0)	2 (22.2)
Leukocytosis	0 (0)	0 (0)	0 (0)	0 (0)	1 (33.3)	1 (33.3)	0 (0)	0 (0)	0 (0)	0 (0)	1 (11.1)	1 (11.1)
Neutropenia	0 (0)	0 (0)	0 (0)	0 (0)	1 (33.3)	1 (33.3)	0 (0)	0 (0)	0 (0)	0 (0)	1 (11.1)	1 (11.1)
Pancreatic carcinoma metastatic^b^	0 (0)	0 (0)	0 (0)	0 (0)	0 (0)	0 (0)	0 (0)	1 (33.3)	1 (33.3)	0 (0)	1 (11.1)	1 (11.1)

^a^ Dose of durvalumab kept constant at 1500 mg every 4 weeks for all cohorts.

^b^ Nodules in injection area defined as metastases by investigator. See the Safety section for details.

DURV, durvalumab; LY, LY2510924.

Four (44.4%) patients experienced at least one TEAE that was ≥grade 3, of which two (22.2%) were assessed as possibly related to the study treatment (one each from the 30 mg and the 40 mg cohort): grade 4 pancreatic carcinoma metastatic and grade 3 leukocytosis; these are the only two treatment-related serious adverse events (SAEs) of the five SAEs that were reported on this trial. The event of “pancreatic cancer metastatic” was reported in one patient who developed several palpable nodules on the thigh that appeared as rim enhancing intramuscular nodules on computed tomography (CT) that could be consistent with metastatic disease.

LY2510924 injections were administered to this specific area on the upper thigh as opposed to the abdomen, which was the recommended location. Given that biopsy of these lesions was not performed, it could not be determined definitively that this represented intramuscular necrotic areas from intramuscular injections of LY2510924 versus metastatic disease. This event was assessed as SAE since no lesions were found outside the area where LY2510924 was administered, although no other similar events have been reported in this study or previously in any other studies involving LY2510924.

### Efficacy

**FIG. 1.**
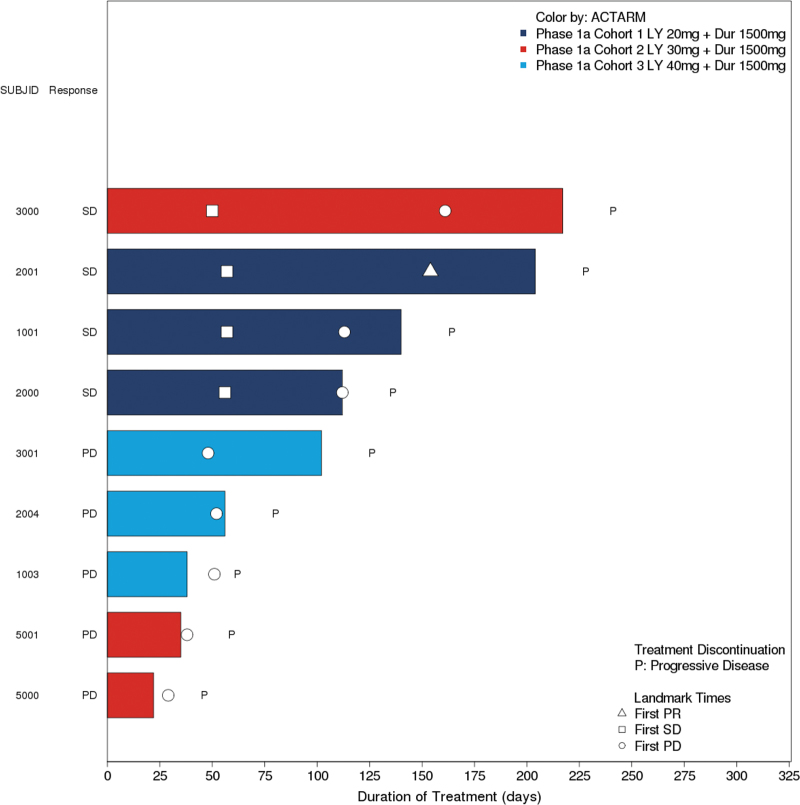
Napoleon plot of treatment duration.

**FIG. 2.**
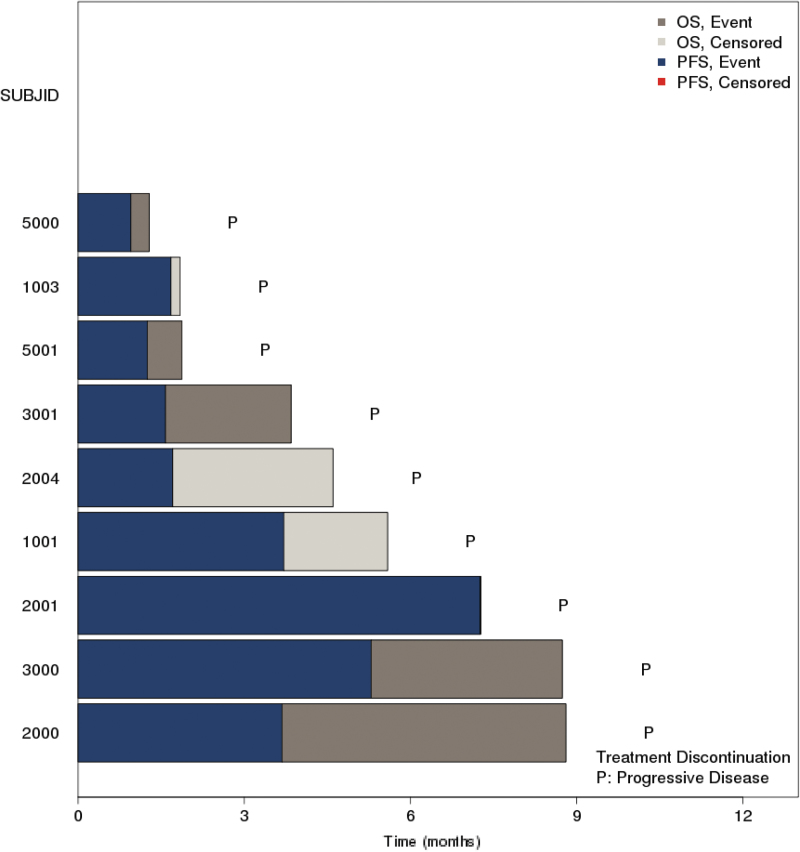
Swim plot of overall survival and progression-free survival.

There were no confirmed complete or partial responses reported in the study. Best overall response of stable disease was observed in four (44.4%) patients. Four patients had stable disease (two patients with pancreatic cancer and one patient with rectal cancer in the 20 mg cohort and one patient with pancreatic cancer in the 30 mg cohort). One of these patients with stable disease with microsatellite stable (20 mg) had an unconfirmed partial response based on a CT-scan at the end of cycle 6 showing a 31% reduction in target lesions. Five patients had progressive disease as best response (2 in the 30 mg cohort and 3 in the 40 mg cohort; Figs. 1 and 2).

### Pharmacokinetic assessments

#### LY2510924

**FIG. 3.**
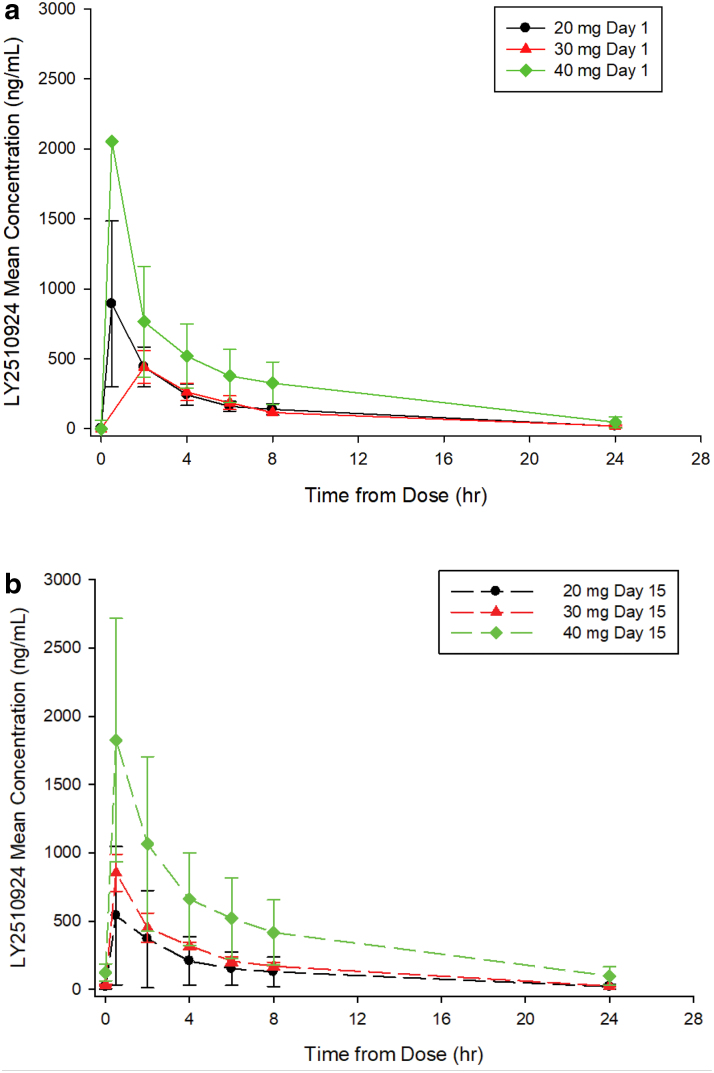
Mean and SD of LY2510924 pharmacokinetic profiles following a 20-, 30-, or 40-mg subcutaneous dose, **(a)** cycle 1 day 1 and **(b)** day 15 coadministered with durvalumab infusion on day 1 of cycle 1. SD, standard deviation.

LY2510924 PK data were available from all nine patients enrolled. The dose levels evaluated were 20, 30, and 40 mg daily injection on a 28-day cycle, dosed continuously. LY2510924 was quickly absorbed after SC administration with a biphasic, dose-dependent decline of LY2510924 serum concentration. Maximum concentration (*C*_max_), *t*_1/2_, and AUC increased with dose, although the 20- and 30-mg dose groups were not well separated based on a small number of patients. LY2510924 mean apparent clearance and volume of distribution were estimated to be 7 L/h and 59 L, respectively. A high level of PK variability was observed, with ∼60% on *C*_max_ and 69% on AUC (Table 3 and Fig. 3). These results were consistent with the LY2510924 PK profile.

**Table 3.** Mean LY2510924 Pharmacokinetic Parameters Following a 20-, 30-, or 40-mg Dose

Geometric mean (CV%)			
LY2510924 dose (mg/day)	20 (n = 3)	30 (n = 3)	40 (n = 3)
Day 1			
*C*_max_ (ng/mL)	746.68 (56)	682.34 (19)	1857.93 (53)
Day 15			
*C*_max_ (ng/mL)	366.67 (169)	847.27 (15)	1678.77 (54)
AUC (0–inf) (ng × h/mL)	1960 (258)	4320 (11)	6210-170000^a^
CL/F (L/h)	10.8 (243)	7.37 (10)	4.10 (52)
*t*1/2^b^ (h)	5.11 (3.11–7.53)	5.98 (5.43–7.15)	7.01–9.14^a^
Vd/F (L)	79.5 (137)	63.6 (16)	36.1–71.2^a^

^a^ *n* = 2, individual values reported.

^b^ Geometric mean (range).

%CV, coefficient of variation; AUC(0–inf), area under the plasma concentration time curve from time zero to infinity; CL/F, apparent clearance; *C*_max_, maximum plasma concentration; *N*, number of patients who received dose; *t*1/2:elimination half-life; Vd/F, apparent volume of distribution.

#### Durvalumab

**FIG. 4.**
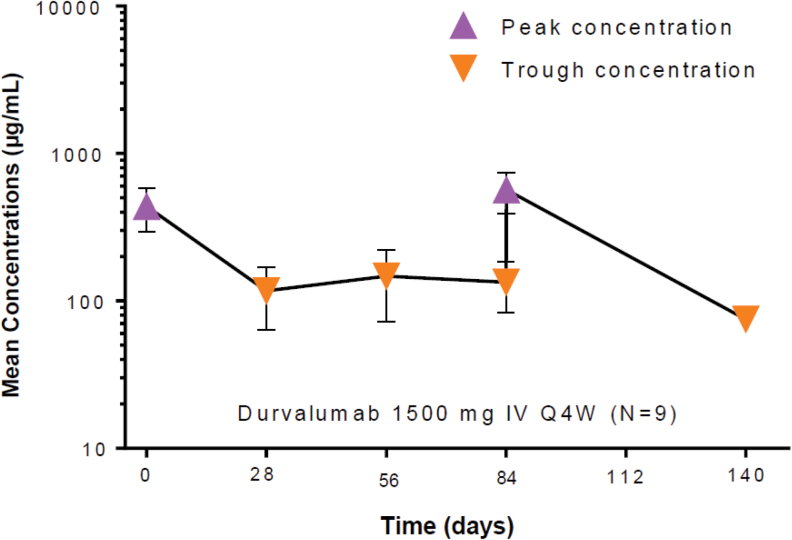
Mean (±SD) serum PK profile of durvalumab following 1500 mg Q4W IV administration. IV, intravenously; PK, pharmacokinetics.

PK data were available for all nine patients following treatment with 1500 mg Q4W of durvalumab administered as an IV infusion over 60 min. Following 1500 mg Q4W durvalumab, the geometric mean (*n*, %CV) of peak concentrations on week 0 and 12 was 418 (*n* = 8, 33.2%) and 549 (*n* = 4, 30.8%) μg/mL, respectively. The geometric mean (*n*, %CV) of trough concentrations on week 4, 8, 12, and 20 was 106 (*n* = 8, 50.3%), 133 (*n* = 4, 58.4%), 126 (*n* = 4, 46.8%), and 74.9 (*n* = 1) μg/mL, respectively (Fig. 4), and was consistent with the durvalumab PK profile.

#### PD assessments

**FIG. 5.**
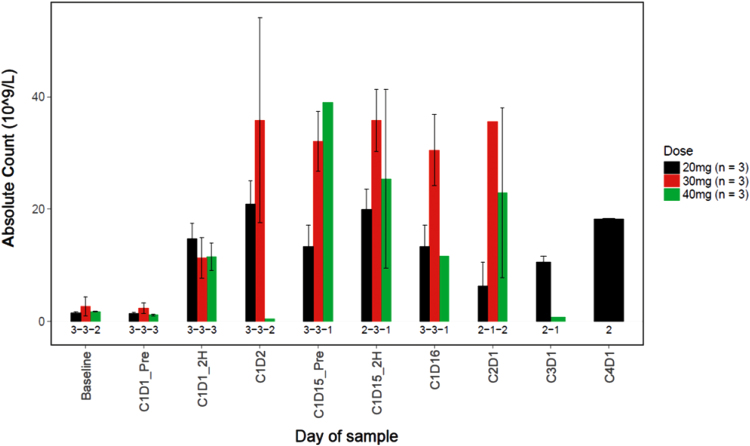
Summary of CD34+ absolute counts (10^9^/L) versus time by dose. At pretreatment, mean CD34+ count was 1.4 (SD = 0.5), 2.3 (SD = 1.6), and 1.1 (SD = 0.3) in the 20, 30, and 40 mg/day dose cohorts, respectively. At C1D15, mean change (increase) from pretreatment in CD34+ count was 4.5 (SD = 2.8), 20.9 (SD = 18.7), and 9.7 (SD = NA) in the 20, 30, and 40 mg/day dose cohorts, respectively.

**FIG. 6.**
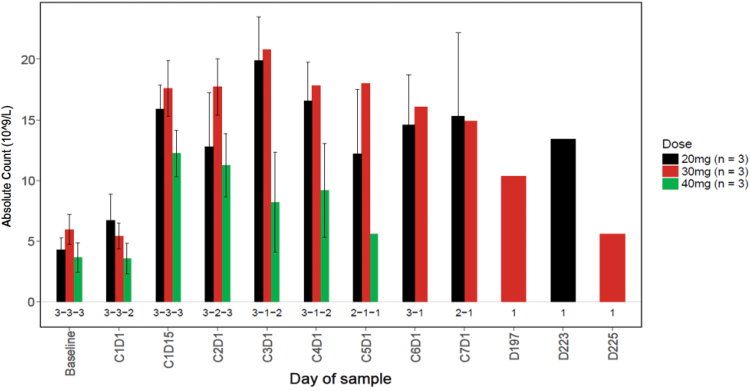
Summary of ANC versus time by dose. At pretreatment, mean ANC was 6.7 (SD = 3.7), 5.4 (SD = 1.8), and 3.6 (SD = 1.8) in the 20, 30, and 40 mg/day dose cohorts, respectively. At C1D15, mean change (increase) from pretreatment in ANC was 2.6 (SD = 0.8), 3.7 (SD = 1.9), and 3.2 (SD = 1.1) in the 20, 30, and 40 mg/day dose cohorts, respectively. ANC = absolute neutrophil count.

**FIG. 7.**
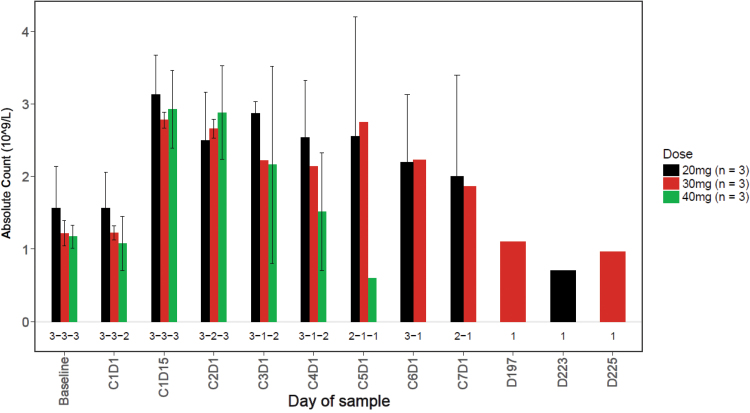
Summary of ALC versus time by dose. At predose, mean ALC was 1.6 (SD = 0.98), 1.2 (SD = 0.3), and 1.2 (SD = 0.29) in the 20, 30, and 40 mg/day dose cohorts, respectively. At C1D15, mean change (increase) from predose in ALC was 2.4 (SD = 1.3), 2.3 (SD = 0.4), and 2.5 (SD = 0.4) in the 20, 30, and 40 mg/day dose cohorts, respectively. ALC, absolute lymphocyte count.

A rapid and sustained increase in CD34+ HSCs was observed in the peripheral blood beginning 2 h following treatment with LY2510924 and durvalumab (Fig. 5). There did not appear to be a relationship between CD34+ counts and LY2510924 dose. The CD34 absolute counts increased in all dose cohorts beginning at cycle 1 day 1 (C1D1) 2 h following treatment and remained increased through C1D16; and in general, the CD34 absolute counts increased in dose cohorts with evaluable data over the course of the study (C2D1, C3D1, and C4D1). The numbers of neutrophils and lymphocytes increased in the peripheral blood in subjects in all dose cohorts by C1D15 and remained increased over the course of the study (Figs. 6 and 7). In addition, LY2510924 and durvalumab mobilized immune cells and triggered an increase in monocytes, T, B, and NK cells (antibodies) in peripheral blood in all dose cohorts at C1D15. No clear dose dependency was observed and data were sparse after cycle 1 (data not shown).

#### PD-L1 expression

PD-L1 protein at baseline was evaluated for six patients (one patient provided both archival tissue and newly obtained biopsy) in three archival tissue sections and four newly obtained biopsies using a fully validated IHC test that determines levels of PD-L1 expression on both tumor cells (TC+) and infiltrating immune cells (IC+). A small set of samples, including fresh and archival biopsies, was analyzed, and no apparent correlation between PD-L1 expression and clinical outcome was observed. The percentage TC+ with PD-L1 membrane staining ranged from 0% to 2%, and four of seven samples did not have PD-L1 expression. PD-L1 TC+ cases included one patient with stable disease and PD, respectively. The percentage IC+ with PD-L1 membrane staining ranged from 0% to 60% and four of seven samples greater than 25% of the immune cells exhibit PD-L1 staining.

## Discussion

LY2510924 is a potent and selective peptide antagonist of CXCR4 with demonstrated antitumor activities in multiple solid tumor xenograft models, and several pre-clinical trials of CXCR4 inhibition have demonstrated significant antitumor efficacy.^7,19,20^ Combination studies with PD-1 inhibition have received attention, given that pre-clinical studies have demonstrated synergy between CXCR4 and PD-1/PD-L1 inhibitors. For example, in a pancreatic cancer model, T cells were typically localized to tumor microenvironment stroma, and the addition of the CXCR4 small-molecule inhibitor AMD3100 led to T cell redistribution and rapid accumulation near tumors, enhancing the antitumor efficacy of an anti-PD-L1 monoclonal antibody combination regimen.^13^

Similarly, in a syngeneic squamous cell carcinoma model, although a single agent of LY2510924 or anti PD-1 antibody showed minimal activity, their combination displayed significant synergy and resulted in complete tumor growth regression. Importantly, LY2510924 mobilized the CD34+ HSCs and leukocytes, increasing these cell counts in circulation.^5,8^ Treatment in these models with CXCR4 antagonists resulted in selective reduction of intratumor regulatory T cells and concomitant increases in T cell-mediated antitumor immune responses, and synergy with PD-1 inhibitors supported the clinical evaluation of LY2510924 and durvalumab in this study.

Based on this phase 1 study, the safety profile for LY2510924 was acceptable for an advanced cancer population. There were no DLTs identified in any of the cohorts. In addition, there were few SAEs, with only two SAEs related to treatment, specifically grade 3 neutropenia and grade 4 “pancreatic carcinoma metastatic.”

The PK parameters for LY2510924 and durvalumab given in combination are similar to those reported in previous studies when given as monotherapy. Within the dose range explored (20–40 mg/day), LY2510924 *C*_max_ and AUC values increased with dose. The PD parameters for LY2510924 given in combination with durvalumab were similar to those in monotherapy as reported previously.^6^ Elevations of similar magnitude and temporal pattern were seen across all leukocyte subsets tested—neutrophils, lymphocytes, monocytes, T, B, NK cells, and CD34+ HSCs. In both the previously reported monotherapy study and this study, a rapid and sustained PD response was observed with treatment as demonstrated by mobilization of leukocytes and stem cells, a clear indication of target modulation. In a monotherapy study of LY2510924, there was a dose relationship between CD34+ counts in peripheral blood at 24 h as the doses increased from 1.0 to 10 mg/day.^6^ However, this response seemed to diminish at doses >10 mg/day, including at the 30 mg/day dose explored in this study. This study did not reveal a clear dose–response for the CD34+ levels at the three doses tested when LY2510924 was given in combination with durvalumab. Similarly, the absolute numbers of neutrophils and lymphocytes increased in the peripheral blood in subjects in all dose cohorts, with a trend toward dose–response relationship only noted between 20 and 30 mg for ANC. This trend is difficult to conclude given the small number of patients and blood collection, as well limited tumor tissue for PD and target modulation evaluation. Despite these limitations, the adverse events did not increase with the LY2510924 dose. Based on safety alone, a dose of 40 mg SC daily LY2510924 in combination with durvalumab is recommended. In future studies, it is imperative to obtain tumor biopsies to confirm the hypothesis that CXCR4 inhibition is associated with increased T cell trafficking to the tumor microenvironment, and further evaluate 40 mg dose as the appropriate dose to accomplish this task. Given the paucity of patients at the RP2D, a larger expansion in pancreatic cancer at the appropriate dose would be necessary to address whether the combination of CXCR4 inhibitor with a PD-1/PD-L1 agent would be efficacious, particularly given the unconfirmed partial response noted in dose escalation.

## Conclusion

The safety profile for LY2510924 was acceptable in the advanced cancer population. Although only one patient had unconfirmed partial response and four patients had stable disease, the PK profiles for LY2510924 and durvalumab, given in combination, were similar to those reported in monotherapy setting. The study confirms the acceptable safety and tolerability of LY2510924 in combination with durvalumab in patients with advanced refractory tumors. The recommended phase 2 dose is 40 mg SC once daily LY2510924 in combination with durvalumab 1500 mg IV Q4W.

## Abbreviations Used

ALC: absolute lymphocyte count
ANC: absolute neutrophil count
AUC: area under curve
CT: computed tomography
CXCR4: chemokine (C-X-C motif) receptor 4
DLT: dose-limiting toxicity
ECOG: Eastern Cooperative Oncology Group
HSC: hematopoietic stem cell
IHC: immunohistochemistry
IV: intravenously
MTD: maximum tolerated dose
PD: pharmacodynamics
PD-1: programmed death 1
PD-L1: programmed death ligand 1
PK: pharmacokinetics
PS: performance status
RCC: renal cell carcinoma
SAE: serious adverse event
SC: subcutaneous
SD: standard deviation
SDF-1: stromal cell-derived factor-1
TEAE: treatment-emergent adverse event
